# Dupilumab for Trichothiodystrophy—Case Report and Review of the Literature

**DOI:** 10.1002/ccr3.72473

**Published:** 2026-04-03

**Authors:** Julia O'Mahony, Cathal O'Connor

**Affiliations:** ^1^ Dermatology South Infirmary Victoria University Hospital Cork Ireland; ^2^ INFANT Research Centre, University College Cork Cork Ireland; ^3^ Paediatrics and Child Health, Cork University Hospital Cork Ireland

**Keywords:** atopic dermatitis, biologic therapy, dupilumab, genodermatosis, ichthyosis, trichothiodystrophy

## Abstract

Trichothiodystrophy (TTD) arises from pathogenic changes in several genes, most of which participate in DNA repair or transcriptional and translational processes. Atopic dermatitis may accompany TTD in a minority of cases. Dupilumab can offer a safe and effective treatment option for severe atopic dermatitis in this population.

## 1 Introduction

Trichothiodystrophy (TTD) is an uncommon autosomal recessive form of congenital ichthyosis (ARCI) characterized by sulfur‐deficient, fragile hair, nail abnormalities, ichthyosiform erythroderma, growth delay, neurodevelopmental concerns, and frequent infections [1]. Variants in several genes, including excision repair cross‐complementation group 3 (*ERCC3*), contribute to its pathogenesis.

## 2 Case History

A newborn of African heritage presented with a collodion membrane, taut and shiny skin, and ectropion. Genetic testing for ARCI identified a paternal pathogenic *ERCC3* variant (c.1841C>A; p.Ser614*) and a maternal variant of uncertain significance (c.1004C>T; p.Ser335Leu), supporting a diagnosis of compound heterozygous TTD.

During the first year of life, the child developed severe atopic dermatitis (AD) that did not respond to emollients or topical corticosteroids. Additional concerns included multiple food allergies, dysphagia felt to be consistent with eosinophilic esophagitis (EE), and recurrent wheezing. Despite the typical association of TTD with very brittle hair, his hair remained relatively strong, which the mother attributed to regular use of hair care products commonly sold in African shops, including formulations with vitamin E, panthenol, shea butter, coconut oil, and other moisturizing or strengthening agents.

## 3 Differential Diagnosis, Investigations and Treatment

**FIGURE 1:**
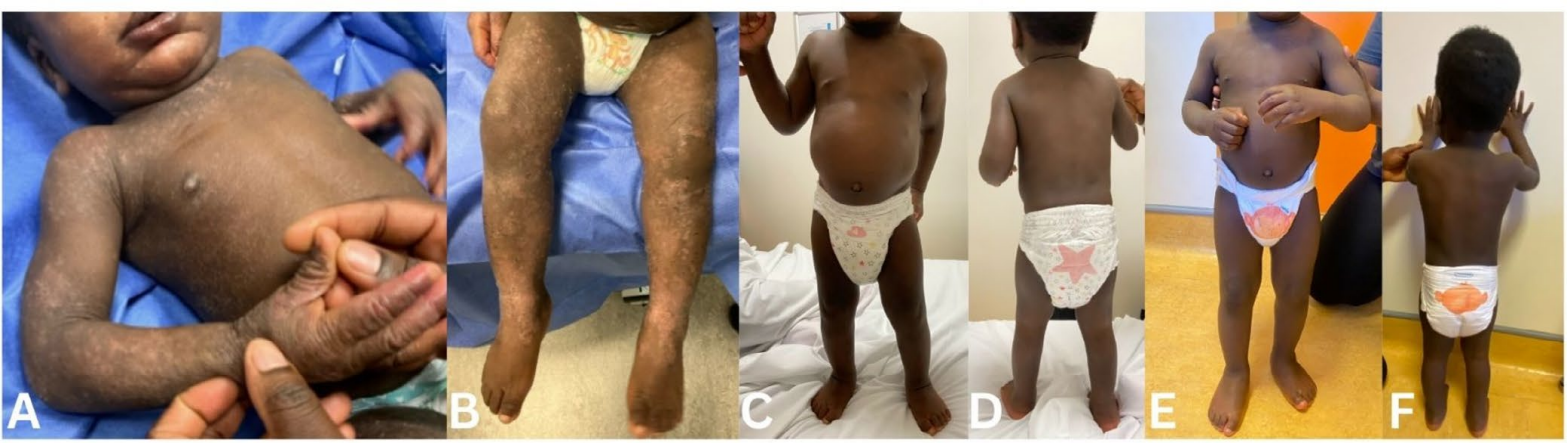
1A–1B prior to administration of dupilumab showing generalized papular eczema, lichenification, and dyspigmentation. 1C–1D after 3 months, showing dramatic improvement with almost clear skin. 1E–F after 6 months, showing sustained response with almost clear skin.

**FIGURE 2:**
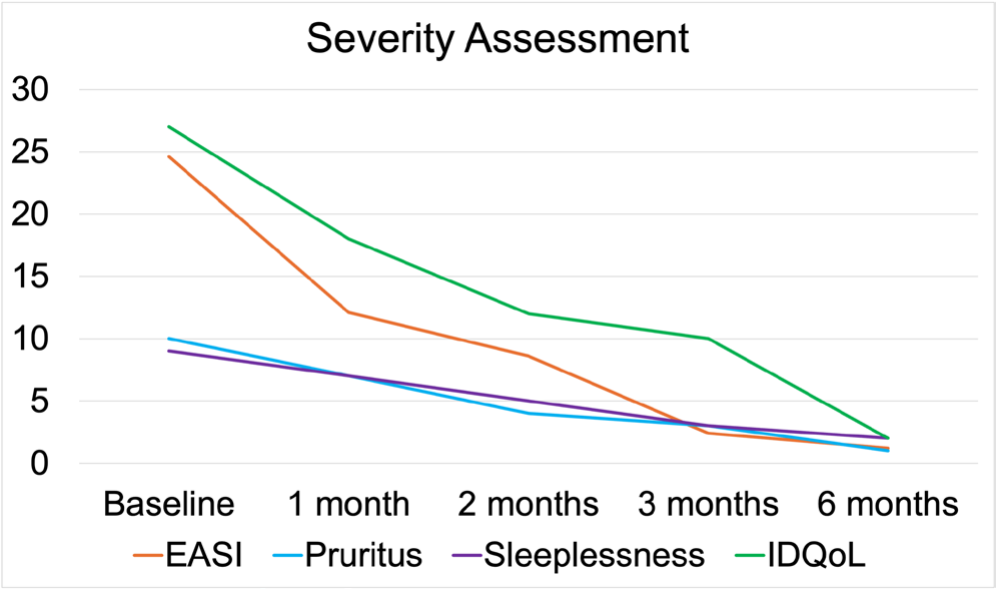
Summary of objective and subjective severity assessment before initiation of dupilumab and for 6 months following initiation of dupilumab. IDQoL = Infants' Dermatitis Quality of Life Index; EASI = Eczema Area and Severity Index.

At 18 months, his eczema area and severity index (EASI) measured 24.6, with widespread papules, lichenification, and excoriations (Figure 1A–1B). His Infants' Dermatitis Quality of Life Index (IDQoL) score was 27/30, with peak itching rated 10/10 and sleeplessness 9/10 (Figure 2). Although dupilumab was licensed for severe AD from 6 months of age, reimbursement for children under 6 years was not yet available in our region. Compassionate access for dupilumab 300 mg every 4 weeks was therefore requested directly from the manufacturer (Sanofi‐Regeneron, Ireland).

## 4 Conclusion and Results

Marked clinical improvement was documented at the four‐week review. By 3 months, his skin was nearly clear, and this benefit persisted through the six‐month assessment (Figure 1C–1F, Figure 2). His EE‐related symptoms also fully resolved. The medication was well tolerated without adverse effects.

## 5 Discussion

Multiple genes have been implicated in TTD, including *ERCC2/XPD*, *ERCC3/XPB*, and *GTF2H5/TTDA*—genes involved in DNA repair and transcription that account for the photosensitive form—and *MPLKIP*, *DBR1*, *RNF113A*, *GTF2E2*, *CARS1*, *TARS1*, *AARS1*, and *MARS1*, which regulate transcription initiation, RNA maturation, and protein translation, and are associated with non‐photosensitive TTD [2]. Around half of individuals with TTD exhibit photosensitivity, but skin cancer risk is not increased [1]. Only a small proportion (about 10%) develop AD [1].

Dupilumab, a monoclonal antibody that blocks interleukin‐4 and interleukin‐13 signaling, is licensed for AD, asthma, and EE in children and is increasingly used off‐label for other dermatologic conditions [3]. Two earlier case reports describe successful dupilumab therapy in children with *ERCC2*‐related TTD and concurrent AD: one child showed major improvement by 3 months with near‐complete clearance by 1 year [4], and another achieved notable improvement by 8 months [5]. Neither report noted drug‐related adverse events [4, 5].

Standard treatments for AD include topical or oral corticosteroids, sedating antihistamines, and systemic immunomodulators such as methotrexate, ciclosporin, mycophenolate mofetil, and azathioprine. These agents carry risks that may be especially relevant in TTD, where growth, neurodevelopment, and immune function can already be compromised. A targeted biologic therapy such as dupilumab therefore represents a particularly suitable therapeutic strategy for children with TTD and severe AD.

## Author Contributions

**Julia O'Mahony:** conceptualization, data curation, formal analysis, investigation, methodology, project administration, resources, software, validation, visualization, writing – original draft, writing – review and editing. **Cathal O'Connor:** conceptualization, data curation, formal analysis, investigation, methodology, project administration, resources, software, supervision, validation, visualization, writing – original draft, writing – review and editing.

## Funding

The authors have nothing to report.

## Conflicts of Interest

The authors declare no conflicts of interest.

## Data Availability

Available on request.
